# Reciprocity in Medical Licensure

**Published:** 1897-07

**Authors:** 


### RECIPROCITY IN MEDICAL LICENSURE.

Dr. William Warren Potter, of Buffalo, president of the National Confederation of State Medical Examining and Licensing Boards, chose this for the subject of his annual address at the seventh annual meeting of that body held at Philadelphia, May 31, 1897. He first paid tribute to the memory of Dr. Perry H. Millard, of St. Paul, then in an introduction reviewed some of the essential points of progress that had been made in State control of medical practice, and finally considered his subject.

### THE PROBLEM.

The most important question now to be discussed pertains to the interstate exchange of licenses, and every friend of State control is interested in establishing this principle. It is one of the objects this confederation is laboring to accomplish but a most difficult problem for solution. A national registration bureau is desirable where legally qualified and reputable physicians may be recorded—physicians whose names appear on this register to be allowed to pass from State to State in the enjoyment of all privileges pertaining to the practice of medicine. Those chiefly agitating the question of reciprocity, however, are specialists who desire to spend profitable vacations at summer resorts and do not relish the idea of taking State examinations in the localities chosen for their holiday practice. Another class of men, compelled by circumstances to change residence, is more deserving of sympathy; they take the examinations uncomplainingly. Shall a State require of its own citizens a compliance with its practice laws while granting to thrifty summer specialists exemption from their operation? As the State laws forbid discrimination against the inhabitants of each there is both a legal and a moral bar to such exemptions.

OBSTACLES TO RECIPROCITY.

Equality of standards for admission to the study and practice of medicine is the only enduring basis on which reciprocity can be established. When the several States adopt a uniform level of preliminaries; a uniform period of collegiate training including uniformity of methods of teaching, and finally, an absolute similarity in the methods of conducting State examinations and granting licenses, then reciprocity will be equitably and permanently established. It is important for the State medical examiners to come to an agreement on these several points that they may act with intelligence on a common platform. The State imposes a postgraduate examination and none should be admitted to it who are not holders of diplomas legally obtained from registered and recognized colleges. It is understood, of course, that there must be established a uniform system of recognizing and registering medical schools in the several States.

THE SOLUTION—LEGISLATIVE ENACTMENTS.

The remedies lie in legislative enactments. Those who most loudly and persistently demand interstate indorsement aim their criticisms at examing boards; whereas, these have nothing to do with the question. The statutes in States that have established licensure prohibit interstate exchange except between such as have equality of standards. The demands of the restless and migratory doctors must be taken to the State legislative halls. Meanwhile, the members of this confederation may assist in bringing the matter to a more speedy conclusion by acquainting their legislatures with the difficulties to be overcome, and by urgently recommending the adoption of such amendments to existing laws as will meet and remove the present defects. Great care must be exercised, however, in the preparation of amendments; the State laws are for the public weal; reciprocity is only for the few. Amendments to existing statutes should be proposed only through State medical examining boards or State medical societies; they are familiar with defects and best know the remedies needed. When legislatures can be persuaded to turn a deaf ear to all amendments that are proposed outside of official sources, it will be a happy day for the friends of State license. The object of this discussion is to divert further criticism of the delay of reciprocity into the proper channel. If legislators could be made to appreciate the fact that public health interests are involved in the question of State license; that every attempt to weaken the principle is a blow at public sanitation; and that higher standards of medical education mean better health for the people, then perhaps it would be easier to obtain and maintain the necessary laws to protect the commonwealths against that kind of ignorance, superstition, or super-refinement that always lurks in the environment of quackery.

